# GPDPLQ_1237_—A Type II Collagen Neo-Epitope Biomarker of Osteoclast- and Inflammation-Derived Cartilage Degradation *in vitro*

**DOI:** 10.1038/s41598-019-39803-0

**Published:** 2019-02-28

**Authors:** Henrik Löfvall, Anna Katri, Aneta Dąbrowska, Morten A. Karsdal, Yunyun Luo, Yi He, Tina Manon-Jensen, Morten H. Dziegiel, Anne-Christine Bay-Jensen, Christian S. Thudium, Kim Henriksen

**Affiliations:** 1 Nordic Bioscience, Herlev, Denmark; 2 Division of Molecular Medicine and Gene Therapy, Lund Strategic Center for Stem Cell Biology, Lund, Sweden; 3 Department of Drug Design and Pharmacology, Copenhagen University, Copenhagen, Denmark; 4 Department of Clinical Immunology, Rigshospitalet, Copenhagen University Hospital, Copenhagen, Denmark

## Abstract

C-telopeptide of type II collagen (CTX-II) has been shown to be a highly relevant biomarker of cartilage degradation in human rheumatic diseases, if measured in synovial fluid or urine. However, serum or plasma CTX-II have not been demonstrated to have any clinical utility to date. Here, we describe the GPDPLQ_1237_ ELISA which targets the EKGPDPLQ↓ neo-epitope, an elongated version of the CTX-II neo-epitope (EKGPDP↓), speculated to be a blood-precursor of CTX-II generated by the cysteine protease cathepsin K. Human osteoclast cartilage resorption cultures as well as oncostatin M and tumour necrosis factor α-stimulated bovine cartilage explant cultures were used to validate GPDPLQ_1237_ biologically by treating the cultures with the cysteine protease inhibitor E-64 and/or the matrix metalloproteinase (MMP) inhibitor GM6001 to assess the potential contributions of these two protease classes to GPDPLQ_1237_ release. Cartilage resorption-derived GPDPLQ_1237_ release was inhibited by E-64 (72.1% inhibition), GM6001 (75.5%), and E-64/GM6001 (91.5%), whereas CTX-II release was inhibited by GM6001 (87.0%) but not by E-64 (5.5%). Cartilage explant GPDPLQ_1237_ and CTX-II release were both fully inhibited by GM6001 but were not inhibited by E-64. No clinically relevant GPDPLQ_1237_ reactivity was identified in human serum, plasma, or urine from healthy donors or arthritis patients. In conclusion, the GPDPLQ_1237_ biomarker is released during osteoclast-derived cysteine protease- and MMP-mediated cartilage degradation *in vitro*, whereas CTX-II release is mediated by MMPs and not by cysteine proteases, as well as from MMP-mediated cartilage degradation under a pro-inflammatory stimulus. These findings suggest that GPDPLQ_1237_ may be relevant in diseases with pathological osteoclast activity and cartilage degradation. Further studies are required to validate the neo-epitope in human samples.

## Introduction

C-telopeptide of type II collagen (CTX-II) is a biomarker of type II collagen degradation, indicative of cartilage degradation. Degradation of joint extracellular matrices (ECMs), including cartilage, is a key feature of multiple types of arthritis, including osteoarthritis (OA) and rheumatoid arthritis (RA). Urinary and synovial fluid (SF) CTX-II, unlike serum CTX-II, is a highly relevant biomarker for OA^1–3^ with some relevance for RA^4^. OA incidence and progression^5,6^, severity^6,7–8^, bone marrow lesions^9^, osteophytes^10^ and pain^11^ have been associated with CTX-II, although pain associations have varied^11^. In RA, CTX-II has been shown to predict disease progression^12–15^, treatment efficacy^13^, bone mineral density (BMD) reduction^16^ and synovitis^16^. However, no studies have been published to date that demonstrate any clinical utility of serum or plasma CTX-II, although it has been demonstrated to be very useful in animal models with experimentally induced joint destruction^17–21^. Additionally, SF and urine sampling have their own drawbacks, particularly in terms of practicality. SF collection is more complicated and inconvenient than collecting either serum, plasma, or urine and is also a more complex biological matrix to work with. Furthermore, both urine and SF analyte concentrations require volume adjustment due to e.g. fluid intake, arthroscopic lavage or joint effusion^3,22^. Urine CTX-II is also known to have diurnal variation^23^, which necessitates a strict urine sampling procedure. As blood is more frequently sampled than urine and SF, a blood-based CTX-II-like biomarker would be more accessible, practical, and reliable for assessing cartilage degradation, if clinical utility could be demonstrated.

CTX-II assays detect C-telopeptide fragments of type II collagen, the major ECM component of articular cartilage, with a neo-epitope^24^ formed by proteolytic cleavage (EKGPDP↓)^25^ during degradation of cartilage by MMPs^26–28^. The neo-epitope has been immunolocalised to areas of cartilage damage, around chondrocytes, areas of vascularization close to the subchondral bone, and the bone–cartilage interface^29^—suggesting possible contribution of osteoclasts to CTX-II. Osteoclasts play important roles in diseases with progressive joint destruction, particularly for bone erosion in diarthrodial joints in RA^30–34^. However, their role in cartilage alterations—such as those seen in OA—is poorly understood, albeit strongly implicated^35^. An elongated CTX-II neo-epitope (EKGPDPLQ↓) has been previously described in patent literature^26–28^, where it has been suggested to be generated by cathepsin K-mediated cleavage of calcified cartilage, to be osteoclast-specific, and to be present in blood while being absent or only present in trace amounts in urine and SF. In the patents, proteases in the kidneys and/or liver were described to potentially remove the C-terminal -LQ of the EKGPDPLQ neo-epitope to generate the urinary forms of the C-telopeptide—EKGPDP or smaller^26–28^. Due to potential differences in generation and/or processing from the EKGPDP neo-epitope, the EKGPDPLQ neo-epitope may be an interesting blood-based biomarker for studies of degenerative joint diseases.

To date no peer-reviewed clinical, *in vivo*, or *in vitro* studies using assays detecting the EKGPDPLQ neo-epitope have been published. In this study we investigated the release of this neo-epitope from non-calcified articular cartilage, in *in vitro* models of cartilage degradation derived from osteoclasts^36^ or inflammation^24^, using the novel competitive GPDPLQ_1237_ enzyme-linked immunosorbent assay (ELISA). The validated assay was then used to test for GPDPLQ_1237_ reactivity in human serum, plasma, and urine.

## Results

### Specificity of the GPDPLQ_1237_ ELISA

The target sequence of the GPDPLQ_1237_ ELISA, _1230_EKGPDPLQ_1237_, was analysed for homology to other human and animal proteins using the NPS@: Network Protein Sequence Analysis PattInProt search against the UniProtKB/Swiss-prot database. The target sequence was found to be unique to the type II collagen alpha 1 chain, and was fully conserved in human, cow, and rat. When allowing for one mismatch in the sequence, it was also conserved in mouse but not in any other animal proteins.

**Figure 1:**
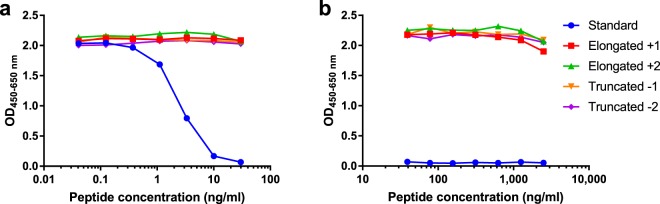
Specificity of the GPDPLQ_1237_ monoclonal antibody. Monoclonal antibody reactivity towards the standard peptide as well as elongated and truncated standard peptides was tested in the competitive GPDPLQ_1237_ ELISA. The OD displacement is shown in the peptide concentration range of the standard curve (**a**) as well as at increased peptide concentrations (**b**). Results are shown as OD_450-650 nm_ as a function of peptide concentration.

The specificity of the competitive GPDPLQ_1237_ ELISA was evaluated by analysing the reactivity towards the standard peptide as well as two elongated and two truncated standard peptides, peptide sequences are described in Table 1. The antibody only reacted with the standard peptide and produced a dose-dependent response (Fig. 1a), increased elongated and truncated peptide concentrations only resulted in minor optical density (OD) displacements (Fig. 1b). These data suggest that GPDPLQ_1237_ is highly specific for the neo-epitope and does not cross-react with the CTX-II neo-epitope, i.e. the Truncated −2 peptide.

**Table 1:** Synthetic peptides used for development and validation of the GPDPLQ_1237_ competitive ELISA.

Peptide identifier	Amino acid sequence
Biotinylated peptide	biotin-EKGPDPLQ
Standard peptide	EKGPDPLQ
Elongated +1 peptide	EKGPDPLQY
Elongated +2 peptide	EKGPDPLQYM
Truncated −1 peptide	EKGPDPL
Truncated −2 peptide	EKGPDP
Immunogenic peptide	KLH-CEKGPDPLQ

Abbreviations: KLH, keyhole limpet haemocyanin.

### Technical validation of the GPDPLQ_1237_ ELISA

Technical validation tests were performed to evaluate the performance of the GPDPLQ_1237_ ELISA. The validation steps and the performance are shown in Table 2. Samples measured linearly in the range 0.8–8.0 ng/ml in dilution recovery tests, samples above 7–8 ng/ml displayed reduced linearity. Samples above 8 ng/ml should be diluted further in assay buffer and measured again. Furthermore, despite obtaining highly reproducible signals in pig, horse, sheep and donkey sera, these signals could not be technically validated by dilution recovery. Hence, sera from these species were only used for determining inter- and intra-assay variation. GPDPLQ_1237_ was, however, abundant in foetal bovine serum (FBS), where it diluted linearly. Human sera, plasma, and urine from healthy donors or RA and OA patients, as well as rat plasma, did not produce sufficient signals for technical validation.

**Table 2:** Technical validation of the GPDPLQ_1237_ competitive ELISA.

Technical validation procedure	GPDPLQ_1237_ performance
Detection range (LLOD–ULOD)	0.7–19.4 ng/ml
Estimated linear range	0.8–8.0 ng/ml
Dilution recovery in linear range^†^	106% (99–119%)
Inter-assay variation^†^	6% (5–8%)
Intra-assay variation^†^	2% (0–2%)
Freeze/thaw recovery (4 cycles)^†^	100% (99–102%)

^†^Percentages are reported as means with ranges shown in brackets.

Abbreviations: LLOD, lower limit of detection; ULOD, upper limit of detection.

### GPDPLQ_1237_ release from bovine articular cartilage explants

**Figure 2:**
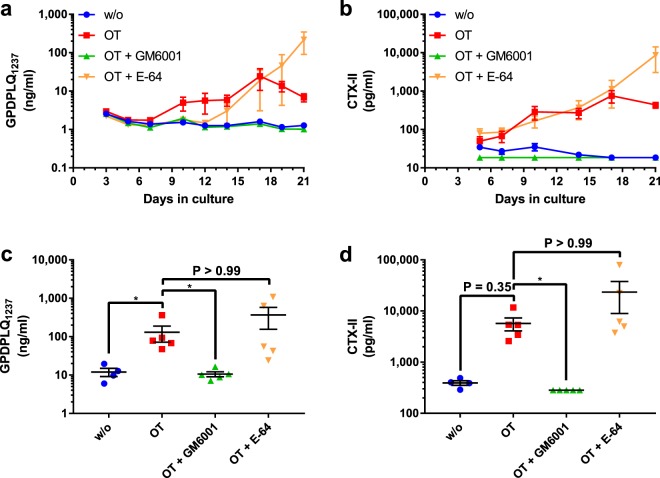
GPDPLQ_1237_ and CTX-II release into BEX supernatants upon stimulation and inhibition of MMP activity. The BEX cultures were untreated (w/o), treated with OSM and TNFα (OT) without protease inhibitors, or OT with the protease inhibitors GM6001 (OT + GM6001) or E-64 (OT + E-64). GPDPLQ_1237_ (**a**,**c**) and CTX-II (**b**,**d**) data from one representative experiment are presented as the mean biomarker levels per group at different time points throughout the experiment (**a**,**b**) and as the AUCs of each individual explant and their means (**c**,**d**). Error bars represent the SEMs. Statistical significance relative to the OT condition in the AUC data is indicated by *P < 0.05.

The generation of the GPDPLQ_1237_ neo-epitope from articular cartilage biopsies undergoing inflammatory degradation was tested in samples derived from bovine cartilage explant (BEX) cultures stimulated with oncostatin M (OSM) and tumour necrosis factor α (TNFα, OT). Although there was substantial variation in GPDPLQ_1237_ induction by OT in terms of magnitude and kinetics between the three performed experiments, the overall inhibition patterns were the same as in the following representative experiment (Fig. 2). GPDPLQ_1237_ data from an additional experiment is shown in Supplementary Fig. S1. The GPDPLQ_1237_ neo-epitope was detected in the OT culture supernatants after approximately 10 days of culture (Fig. 2a), but GPDPLQ_1237_ levels remained at baseline levels when adding the broad-spectrum MMP inhibitor GM6001 to the culture. The OT response was not inhibited by the cysteine protease inhibitor E-64, albeit with considerable variation between explants, but there was a delay in GPDPLQ_1237_ release in the E-64 condition relative to the OT condition and the E-64 condition had higher GPDPLQ_1237_ levels than the OT condition at the final time points, findings that were also present in additional experiments (Supplementary Fig. S1). The apparent decrease in GPDPLQ_1237_ levels in the OT condition at the final time points compared to earlier time points (Fig. 2a) was, however, not a consistent finding (Supplementary Fig. S1). The GPDPLQ_1237_ area under the curve (AUC) analysis (Fig. 2c) demonstrates that OT induced a statistically significant increase in GPDPLQ_1237_ levels (129.9 ± 58.6 ng/ml, P = 0.034) that was fully inhibited by GM6001 (10.6 ± 1.6 ng/ml, P = 0.015), but not by E-64 (367.2 ± 212.2 ng/ml, P > 0.999). Similar results were also found in additional experiments (Supplementary Fig. S1). CTX-II release, measured at a selection of relevant time points, followed a very similar pattern to that of GPDPLQ_1237_ release (Fig. 2b); OT induced CTX-II release while GM6001, but not E-64, inhibited the CTX-II induction to baseline levels. Unlike GPDPLQ_1237_ release, CTX-II release in the E-64 condition did not appear to have a delayed onset relative to the OT condition. Increased CTX-II levels were observed in the E-64 condition at late time points, similarly to the GPDPLQ_1237_ data. The CTX-II AUC analysis (Fig. 2d) reveals that OT induced a marked increase in CTX-II levels, albeit not statistically significant (5695.0 ± 1608.0 pg/ml, P = 0.345), that was fully inhibited by GM6001 (283.2 ± 0.0 pg/ml, P = 0.010), but not by E-64 (23309.0 ± 14381.0 pg/ml, P > 0.999).

### GPDPLQ_1237_ release from articular cartilage resorption

**Figure 3:**
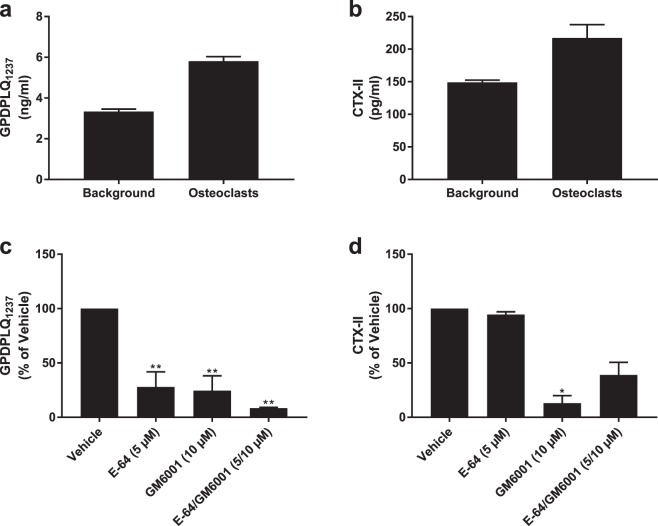
Osteoclast-derived GPDPLQ_1237_ and CTX-II release from bovine articular cartilage. Background biomarker levels of GPDPLQ_1237_ (**a**) and CTX-II (**b**) measured in media from cultures with only cartilage (Background, n = 2) and cultures with osteoclasts and cartilage (Osteoclasts, n = 6) in one representative resorption assay, presented as means with SEMs. Background levels were subtracted from the GPDPLQ_1237_ and CTX-II levels in each individual resorption assay followed by normalisation to the vehicle condition. The normalised data were averaged followed by pooling of the data, resulting in n = 2 for E-64/GM6001 and n = 3 for other conditions where n is the number of resorption assays featuring the condition, and the background-reduced normalised GPDPLQ_1237_ (**c**) and CTX-II (**d**) levels are presented as the means of all resorption assay means where error bars represent the SEMs. Statistical significance relative to the vehicle condition in the background-reduced data is indicated by *P < 0.05 and **P < 0.01.

The release of GPDPLQ_1237_ from human osteoclasts cultured on bovine non-calcified articular cartilage was measured in three individual resorption assays. As demonstrated in a representative experiment, the absolute GPDPLQ_1237_ levels (Fig. 3a) markedly increased in the supernatants of cultures with osteoclasts compared to background levels in cultures without osteoclasts, as did CTX-II levels (Fig. 3b). The background measurements were subtracted from the osteoclast measurements from each assay, and the background-subtracted data were subsequently normalised to the vehicle condition mean. The normalised data from each condition were averaged and pooled with the data from the other resorption assays to generate the final data set, where n corresponds to the number of resorption assays per condition. The background-subtracted GPDPLQ_1237_ release (Fig. 3c) was inhibited by both E-64 (72.1 ± 14.0% inhibition, P = 0.007) and GM6001 (75.5 ± 13.7% inhibition, P = 0.006) compared to the vehicle condition. The GPDPLQ_1237_ levels were reduced further by E-64/GM6001 combined (91.5 ± 0.7% inhibition, P = 0.004), although this was not statistically significant compared to E-64 or GM6001 alone (P = 0.659 and P = 0.769). As expected, GPDPLQ_1237_ was not detectable above background levels in supernatants from resorbed cortical bone (data not shown). CTX-II release (Fig. 3d) in the cartilage resorption cultures was inhibited by GM6001 (87.0 ± 6.9% inhibition, P = 0.019) but not by E-64. E-64/GM6001 combined did not result in further inhibition of CTX-II release (61.1 ± 11.6% inhibition, P = 0.246). These findings indicate that the release of GPDPLQ_1237_ and CTX-II neo-epitopes from articular cartilage undergoing osteoclast-mediated degradation are not mediated by the same proteases; while we demonstrated that both MMPs and cysteine proteases contribute to GPDPLQ_1237_ release during osteoclastic cartilage resorption, only MMPs contributed to CTX-II release.

### Immunolocalization of the GPDPLQ_1237_ neo-epitope

**Figure 4:**
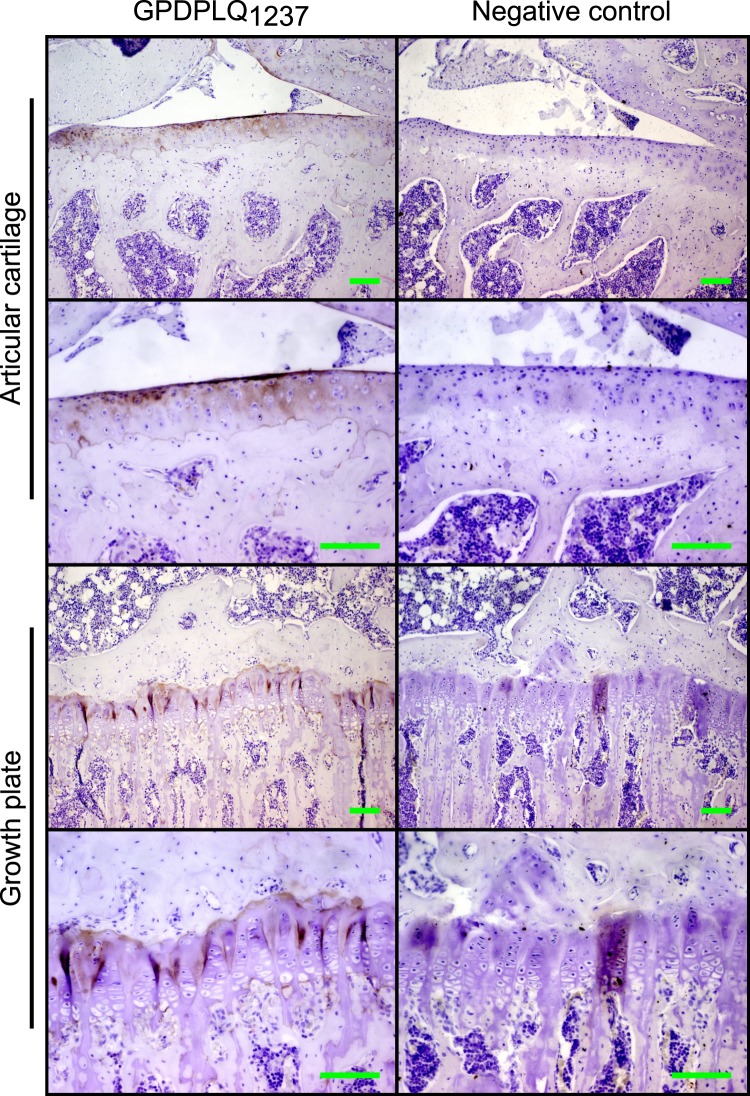
Immunolocalization of the GPDPLQ_1237_ neo-epitope in rat tibia. Representative micrographs of rat tibial articular cartilage and growth plate stained with the NB427-5G11-4W3 GPDPLQ_1237_ (GPDPLQ_1237_) monoclonal antibody (brown) or a negative control antibody, counterstained with Mayer’s haematoxylin (purple). Micrographs were obtained using a 10x (first and third row) and a 20x (second and fourth row) objective. Scale bars represent 100 µm.

Tibia obtained from young healthy Lewis rats were used to immunolocalise the GPDPLQ_1237_ neo-epitope. GPDPLQ_1237_ staining was observed at the articular cartilage and the growth plate, while no staining was observed when using a negative control antibody (Fig. 4). The articular cartilage stained for GPDPLQ_1237_ in the ECM and in pericellular spaces throughout all cartilage layers, albeit at varying intensity, while the growth plates stained mainly between the columns of proliferating cells—not in the immediate pericellular ECM—and at the proximal bone–cartilage interface. Intermittent growth plate staining was also observed in the ossification zone.

## Discussion

There is an unmet need for novel biomarkers of cartilage degradation for rheumatic diseases, and for blood-based biomarkers in particular. The EKGPDPLQ neo-epitope has been previously described in patent literature^26–28^, but to our knowledge no peer-reviewed articles measuring EKGPDPLQ release from cartilage, calcified or non-calcified, have been published. In patent literature, the EKGPDPLQ neo-epitope has been described to represent the degradation of calcified cartilage by cathepsin K^26–28^, if analysed in blood. However, using the competitive GPDPLQ_1237_ ELISA we could quantify both cysteine protease- and MMP-mediated release of the EKGPDPLQ neo-epitope from different models of non-calcified cartilage degradation *in vitro*.

In our study, we detected MMP-derived GPDPLQ_1237_ release from non-calcified cartilage in a model of inflammatory cartilage degradation. In the BEX OT model, the GPDPLQ_1237_ protease profile—with inhibition by GM6001 and no inhibition by E-64—and kinetics were similar to those of CTX-II. These CTX-II results also closely resembled previously reported CTX-II^37,38^ and C2M^39^ data in the BEX OT model. The C2M neo-epitope, like CTX-II, is generated from type II collagen degradation predominantly by MMPs^39^. C2M has previously been shown to be able to distinguish between healthy subjects and patients or between subsets of patients with arthritic diseases, such as OA^39,40^, RA^41–43^ and ankylosing spondylitis^44,45^, and is therefore a relevant biomarker for assessing type II collagen resorption in *in vitro* models. Interestingly, CTX-II release is typically increased by E-64 treatment in the BEX OT model^37,38^, whereas C2M release is not increased to a statistically significant degree^39^, but we did not detect any significant differences in GPDPLQ_1237_ or CTX-II release between the OT and the OT+ E-64 conditions. The overall effect of E-64 on both GPDPLQ_1237_ and CTX-II AUC release was highly variable and not statistically significant, indicating that cysteine proteases did not have a net effect that played any major role in release of either biomarker in this model, although it is possible that specific cysteine proteases may contribute differently. The lack of overall inhibition of GPDPLQ_1237_ release by E-64 is likely due to low cysteine protease activity in this model, even though cathepsin K induction has previously been demonstrated in the BEX OT model by immunohistochemistry^37^. Similarly to our findings, the cathepsin K-specific type II collagen neo-epitope C2K77 has been reported to not be detectable above background levels in OT-stimulated equine cartilage explants^46^, indicating that cathepsin K-mediated type II collagen degradation—and potentially the contribution of other cysteine proteases as well—is low in this model.

The reason for the apparent delayed release of GPDPLQ_1237_ in the E-64 condition as well as the increased GPDPLQ_1237_ and CTX-II levels at late time points, compared to the OT condition, remains unclear. Parts of these effects are likely explained by the large inter-explant variation that is common for this model system, although we did observe both the delayed GPDPLQ_1237_ onset and the elevated biomarker levels at final time points in several experiments. The delay in GPDPLQ_1237_ release did not appear to affect overall biomarker levels over the culture period used in this experiment, and the biological relevance of this putative delay is therefore uncertain. Interestingly, no corresponding delay was observed in CTX-II release upon E-64 treatment, suggesting that there might be a difference between GPDPLQ_1237_ and CTX-II in the kinetics of biomarker release at intermediate time points that may be related to cysteine protease activity. Speculatively, the delay may be a result of E-64 temporarily inhibiting early cysteine protease-mediated biomarker release, which is detectable by the GPDPLQ_1237_ assay but not by the CTX-II assay, but the inhibition ultimately fails and results in rapid onset of cysteine protease-mediated cartilage degradation and GPDPLQ_1237_ release.

The increased GPDPLQ_1237_ release at late time points in the E-64 condition may potentially be mediated by inhibition of cathepsin B. Cathepsin B is a dipeptidyl carboxypeptidase^47^ that is upregulated in interleukin-1 (IL-1)^48^ and TNFα/IL-1β-stimulated^49^ chondrocytes and may potentially process the EKGPDPLQ neo-epitope into the EKGPDP neo-epitope. Although cathepsin B is known to generate CTX-II during *in vitro* cleavage—while cathepsins D, K, L and S do not—and may even be able to degrade CTX-II neo-epitopes to some extent^50^, it is unclear how much cathepsin B actually contributes to CTX-II release in the BEX OT model. As demonstrated in our study, cysteine protease inhibition with E-64 does not reduce CTX-II release in the BEX OT model but rather appears to have no effect or to increase it, as has been previously reported^37,38^, indicating that the contribution of cathepsin B to type II collagen degradation, CTX-II release, GPDPLQ_1237_ degradation, and/or CTX-II degradation in this model is modest compared to the net neutral or positive effects that cysteine protease inhibition appears to have on CTX-II levels at late time points. Furthermore, the overall lack of effect of E-64 in GPDPLQ_1237_ and CTX-II AUC data and the increased release of both GPDPLQ_1237_ and CTX-II at late time points suggest that the late increase of GPDPLQ_1237_ levels is not a result of inhibited conversion of the GPDPLQ_1237_ epitope into CTX-II by cysteine proteases—as E-64 treatment only resulted in delayed GPDPLQ_1237_ induction while it did not increase and decrease overall GPDPLQ_1237_ and CTX-II release, respectively. While cysteine proteases do not appear to have a significant net effect on overall GPDPLQ_1237_ and CTX-II release, cathepsin B may still convert GPDPLQ_1237_ into CTX-II in this model. If cathepsin B mediates both GPDPLQ_1237_ and CTX-II degradation at similar rates it is possible that neither of these effects would be detectable during cysteine protease inhibition, or indeed cathepsin B inhibition. The potential role of cathepsin B in conversion of GPDPLQ_1237_ into CTX-II is interesting and could be investigated further using cathepsin B-specific inhibitors such as CA074^51^, potentially in BEX cultures stimulated with IL-1, but this was considered to be outside the scope of the current study as cysteine protease inhibition did not appear to have substantial impact on the overall release of either biomarker in the BEX OT model.

As for the observed decline in biomarker release in the OT condition at very late time points, this is similar to previously reported results in other type II collagen neo-epitopes at very late time points, both in OT and OT+ E-64 conditions^38,39^, but it was not a consistent finding in our study, underscoring the somewhat variable nature of cartilage explant cultures. As it was not consistent and was observed with both GPDPLQ_1237_ and CTX-II, albeit to a lesser extent with CTX-II, the decline is unlikely to be a result of interconversion of the neo-epitopes by cathepsin B or to represent important changes to type II collagen degradation over time. The decline may rather be related to e.g. destruction of the cartilage or depletion of the ECM, as has been previously described in this model^37^, or the substantial inter-explant variation that is often observed in the BEX OT model.

In our model of osteoclastic cartilage resorption, GPDPLQ_1237_ release was found to be mediated by both cysteine proteases and MMPs. Cathepsin K is the predominant cathepsin in osteoclasts^52,53^ and it has also been demonstrated to be crucial for type I collagen degradation by osteoclasts during normal bone resorption^52–56^. Furthermore, the cathepsin K-specific antiresorptive odanacatib inhibits the majority of CTX-I release (approximately 80% inhibition) during bone resorption *in vitro*^55,56^ with an effect size similar to that of E-64^57^. Osteoclasts are also thought to utilize several other cathepsins and cysteine proteases for bone resorption^58,59^, but cathepsin K is clearly essential for normal resorptive function and type I collagen resorption even though MMPs can compensate to some extent when cathepsin K is inhibited^60^. Accordingly, it is likely that the cysteine protease-mediated GPDPLQ_1237_ release from cartilage is mediated at least in part by cathepsin K, but the precise cysteine proteases underlying GPDPLQ_1237_ release during cartilage resorption would need to be verified using specific inhibitors, like odanacatib, or cathepsin K-deficient osteoclasts. Contrary to GPDPLQ_1237_ release, CTX-II release from cartilage resorption was mediated by MMPs but not by cysteine proteases. This is similar to previous studies of CTX-II release from cartilage digested by MMPs or cathepsins D, K, L and S *in vitro*^50,61^, although CTX-II has been shown to be generated by cathepsin B^50^. Thus, GPDPLQ_1237_ appears to be a biomarker that is generated through direct osteoclastic cartilage resorption mediated by cysteine proteases—likely in part by cathepsin K—and MMPs whereas CTX-II release is primarily mediated by MMPs in this model, similarly to previous studies of C2M release in this model^36^.

The potential contribution of MMPs, cysteine proteases and articular cartilage to GPDPLQ_1237_ neo-epitope levels may differ *in vivo*. GPDPLQ_1237_ levels in human serum, plasma and urine, obtained from healthy donors, OA patients, or RA patients, or rat plasma were too low to perform technical validation of the assay in these matrices. Further work would be required to validate the neo-epitope’s relevance in human samples. Although we do not know the reason underlying the lack of substantial GPDPLQ_1237_ signals in serum, plasma, and urine in this study, it may be due to insufficient sensitivity of the ELISA or processing of the EKGPDPLQ neo-epitope into the EKGPDP neo-epitope^26–28^, followed by excretion, resulting in low levels or transient presence of the EKGPDPLQ neo-epitope in serum and plasma. This would require further testing and validation, potentially on more sensitive assay platforms or using a redesigned assay that may allow for improved sensitivity towards the analyte. However, preliminary attempts to change the assay buffer or assay design, using e.g. a sandwich ELISA instead of a competitive ELISA, did not improve sensitivity towards the analyte in serum or plasma. Alternatively, GPDPLQ_1237_ may be measured in SF which may be a more biologically relevant matrix, although the patents proposed otherwise^26–28^. SF measurements were outside the scope of the current study as we aimed to develop a blood- or urine-based biomarker, due to the limited availability of SF and its invasive sampling method, and GPDPLQ_1237_ presence in SF would need to be investigated further. Other elongated or truncated CTX-II neo-epitopes^26–28^ may also be relevant targets in the search for CTX-II-like neo-epitopes in blood.

Despite the lack of GPDPLQ_1237_ in human and rat body fluids, our immunohistochemical analysis of GPDPLQ_1237_ presence in rat tibia suggests that the neo-epitope can be generated through naturally occurring cartilage turnover, at least in the joints of young adult rats. GPDPLQ_1237_ was observed in the articular cartilage ECM in healthy Lewis rats. CTX-II is primarily present in articular cartilage during disease, e.g. collagen-induced arthritis, in rats^18,62^ but it has also been described in some healthy rats^62^. In our study, GPDPLQ_1237_ was present at various levels in all layers of the articular cartilage ECM in healthy rats, unlike CTX-II which is only present in some healthy rats^62^, which suggests that articular cartilage GPDPLQ_1237_ may be a feature of physiological cartilage turnover in rats, whereas CTX-II is a better indicator of pathology. Additionally, the articular cartilage GPDPLQ_1237_ staining patterns differ from those reported for CTX-II; CTX-II stains the superficial matrix in the upper zone as well as around round and flat chondrocytes in the upper and deep zones^62^, although it is unclear which treatments the rats that were used in the reported illustrations were subjected to in this report. Similar CTX-II staining patterns have also been reported in human OA knees^29^. Interestingly, CTX-II frequently stains the bone–cartilage interface in these tissues^29^, but human growth plate stainings were not available for comparison to our GPDPLQ_1237_ findings. When examining the rat growth plates, we found that GPDPLQ_1237_ primarily stained the proximal bone–cartilage interface and the ECM between the columns of proliferating chondrocytes, but not the pericellular matrix. We also observed intermittent GPDPLQ_1237_ staining in the ossification zone, where osteoclasts degrade the calcified cartilage during endochondral ossification^63^. Growth plates in both healthy rats and arthritic rats can stain positive for the CTX-II neo-epitope, but they do so in a different pattern than GPDPLQ_1237_^62^. CTX-II staining was reported primarily in the pericellular ECM around the chondrocytes in the illustrative growth plate staining^62^. Although the GPDPLQ_1237_ and CTX-II staining patterns clearly differ, the presence of both GPDPLQ_1237_ and CTX-II at healthy rat growth plate suggests that they may both be generated as part of endochondral ossification during bone growth. This conclusion is further supported by both GPDPLQ_1237_ and CTX-II^17^ being abundant in FBS, as endochondral ossification is an important part of bone development during foetal development. Similarly, both CTX-II and C2M are high in serum from one-month-old rats but decrease significantly over time as they age^64^.

Other type II collagen neo-epitope antibodies, such as C2M and C2K77 have also been described to be present in OA cartilage. C2M is known to be present in both non-calcified and calcified cartilage beneath cartilage surface irregularities, surface erosions, and cartilage lesions^39^. Similarly, C2K77 staining is increased in equine OA cartilage sections with focal cartilage degeneration and partial thickness fissures^46^, as well as in areas of proteoglycan loss^65^. C2K77 staining has also been demonstrated in human knee OA cartilage lesions where it mostly stained the matrix around chondrocytes in the lesional area of the tissue, whereas little staining was observed in non-lesion areas or with a negative control antibody^65^. These immunohistochemical stainings of the type II collagen neo-epitopes CTX-II, C2M and C2K77 demonstrate how they can be used to further our understanding of disease processes in different parts of joints, but descriptions of C2M, C2K77 and CTX-II stainings of healthy cartilage are limited. In our study we observed substantial GPDPLQ_1237_ staining in both cartilage and growth plates in healthy rat specimen, which would make it difficult to make any conclusions on GPDPLQ_1237_ generation in diseased specimen. However, our immunohistochemical analysis does demonstrate that GPDPLQ_1237_ is generated biologically and is present in rat tissues, even though we were not able to measure and validate GPDPLQ_1237_ in rat plasma. Immunohistochemical studies using human tissues would be of interest to investigate if GPDPLQ_1237_ staining is also a feature of mature human cartilage or if it is specific to diseased human cartilage.

In conclusion, the results of our study indicate that the GPDPLQ_1237_ neo-epitope—EKGPDPLQ—is a non-calcified cartilage degradation biomarker that can be generated during osteoclastic resorption and inflammatory degradation by cysteine proteases and/or MMPs *in vitro*, and during physiological cartilage degradation in rats. GPDPLQ_1237_ is a promising biomarker for studies of cartilage degradation *in vitro*, but additional work is required to validate the relevance of the neo-epitope *in vivo*.

## Methods

### Target selection and peptide sequences

The GPDPLQ_1237_ target sequence, as described in patents^26–28^, is a C-terminal neo-epitope formed by proteolytic cleavage of type II collagen: _1230_EKGPDPLQ↓YMR_1240_. A sequence of 8 amino acids adjacent to the cleavage site (_1230_EKGPDPLQ↓_1237_) was chosen as the target. The sequence was analysed for homology to other human and animal proteins using NPS@: Network Protein Sequence Analysis PattInProt search^66^ with the UniProtKB/Swiss-prot database.

Synthetic peptides (GenScript, Piscataway, NJ) were used for monoclonal antibody production and validation of the ELISA. An overview of the peptides used is shown in Table 1. A biotinylated peptide (biotin-EKGPDPLQ) was used as a coating peptide for streptavidin-coated microtiter plates. Standard curves were generated using the standard peptide (EKGPDPLQ). The specificity of the antibody was tested using two elongated and two truncated standard peptides, that have been previously described^26–28^. An immunogenic peptide was generated by covalent cross-linkage of a standard peptide with an added N-terminal cysteine residue to keyhole limpet haemocyanin (KLH) carrier protein using succinimidyl 4-(*N*-maleimidomethyl)cyclohexane-1-carboxylate (SMCC, Thermo Scientific, Waltham, MA).

### Generation of monoclones

Four to six-week-old female Balb/C mice were housed (five mice per cage, standard wood chips bedding enriched with huts, nesting material and sticks) at the Nordic Bioscience (Beijing) animal facility (21–23 °C, 55–65% relative humidity, 12 hours light/dark cycle) with *ad libitum* access to food and water. The mice were immunised by subcutaneous injection of 200 μl emulsified antigen containing 60 μg immunogenic peptide mixed with Freund’s complete adjuvant (Sigma-Aldrich, St. Louis, MO). Repeated immunizations with 30 μg immunogenic peptide mixed with Freund’s incomplete adjuvant (Sigma-Aldrich, St. Louis, MO) were performed in 2-week intervals until stable serum titres were obtained. The mice with the highest titre and highest reactivity towards the target, tested in competitive ELISAs using the biotinylated, standard, elongated and truncated peptides, were allowed to recover for four weeks followed by a booster injection of 50 μg immunogenic peptide in 100 μl 0.9% NaCl solution, administered intraperitoneally. When serum titres were high and stable, the mice were euthanised by concussion followed by cervical dislocation and isolation of their spleens. Hybridoma cells were generated through fusion of isolated splenocytes with SP2/0 myeloma cells as previously described^67^. Limited dilution procedures were used to generate monoclonal cultures.

### Specificity of monoclonal antibodies

The reactivity of the monoclonal supernatants was evaluated in a competitive ELISA by testing for OD displacement using the standard peptide and lack of displacement by elongated and truncated standard peptides, using 4 ng/ml biotinylated peptide on streptavidin-coated microtiter plates (Roche, Basel, Switzerland, cat. 11940279). The clones with the best OD displacement profiles (data not shown) were used to generate antibodies that were purified using protein G columns according to the manufacturer’s instructions (GE Healthcare Life Sciences, Little Chalfont, UK, cat. 17-0404-01).

### GPDPLQ_1237_ assay protocol

Optimal assay buffer, incubation times, temperature, and concentrations of antibody and peptides were determined and the finalised GPDPLQ_1237_ competitive ELISA protocol is described below. A 96-well streptavidin plate was coated with 100 µl of 625 pg/ml biotinylated peptide dissolved in coating buffer (10 mM phosphate-buffered saline [PBS] with 1% bovine serum albumin and 0.1% Tween 20 [PBS-BTB], 8 g/l NaCl, pH 7.4) and incubated for 30 minutes at 20 °C. 20 μl of peptide calibrators prepared as a 3-fold dilution series of 30.03 ng/ml standard peptide, controls or samples were added to the appropriate wells followed by 100 μl of antibody NB427-5G11-4W3 dissolved in assay buffer (50 mM PBS-BTB, 8 g/l NaCl, pH 7.4) to a concentration of 526 ng/ml and incubation for 20 ± 1 hours at 4 °C. 100 µl of horseradish peroxidase (HRP)-conjugated rabbit anti-mouse secondary antibody (Jackson ImmunoResearch Laboratories, West Grove, PA, cat. 315-035-045) dissolved in assay buffer to a concentration of 160 ng/ml was added to the wells and incubated for 1 hour at 20 °C. All the above incubation steps included shaking at 300 rpm and were followed by five washes in washing buffer (25 mM Tris, 50 mM NaCl, 0.1% Tween 20, pH 7.2). Finally, 100 μl 3,3′,5,5′-tetramethylbenzidine (TMB, Kem-En-Tec Diagnostics, Taastrup, Denmark, cat. 4380) was added and incubated for 15 minutes at 20 °C in the dark with shaking at 300 rpm followed by the addition of 100 μl of 0.18 M H_2_SO_4_ to stop the reaction. The absorbance was measured at 450 nm with 650 nm as a reference on a VersaMax (Molecular Devices, Sunnyvale, CA) and a standard curve was plotted using a 4-parametric mathematical fit model in SoftMax Pro v.6.3 for Windows (Molecular Devices, Sunnyvale, CA). Cartilage resorption supernatants were diluted 1:1–1:5 as needed, BEX supernatants were diluted approximately 1:1–1:4000 as needed, serum, plasma, and urine samples were measured undiluted. Any samples measuring below the lower limit of detection (LLOD) were assigned the LLOD as their result.

### Technical validation

The technical performance of the ELISA was validated in the following tests: LLOD, upper limit of detection (ULOD), measuring range (LLOD–ULOD), linear range and dilution recovery, inter- and intra-assay variation, specificity, and analyte freeze-thaw stability. The LLOD was determined from three independent runs of 68 samples of the zero standard (i.e. assay buffer). The LLOD was calculated within each run as the mean + three standard deviations and the final LLOD was calculated as the mean of the three runs. The ULOD was determined from five independent runs of the highest standard, measured in duplicates. The mean concentrations of the duplicates, as calculated from the standard curve, were used to calculate a ULOD defined as the mean of the five measurements - three standard deviations. The linear range of the assay was estimated from dilution recovery tests by performing 2-fold dilution series of a heat-inactivated FBS sample and a culture supernatant from a BEX OT culture and calculating the percentage dilution recovery against different starting dilutions. The linear range was considered to be the longest range of measured concentrations that, when adjusted for dilution, had a dilution recovery within 100 ± 20% of the adjusted concentration of the starting dilution. Inter- and intra-assay variation was determined from five independents runs of six positive control samples—diluted FBS, diluted BEX OT, and sera from pig, horse, sheep and donkey measured in duplicates—and the inter- and intra-assay coefficients of variation (CVs) of each sample were calculated. Specificity was tested using elongated and truncated standard peptides (Table 1). The analyte stability was determined for one FBS sample and one BEX OT sample for up to four freeze-thaw cycles.

### BEX cultures

BEXs were harvested by dissecting the outermost layer of articular cartilage from bovine knee joints and were cultured as previously described^68^. In brief, cartilage explants were placed in 96-well plates and incubated at 37 °C with 5% CO_2_ under serum-free conditions (n = 4–5 per condition). Each explant was cultured in 200 μl DMEM/F-12 medium for 21 days, with medium changes every 3–4 days, under one of the following conditions: without cytokines, with 10 ng/ml OSM and 20 ng/ml TNFα (OT) to stimulate MMP activity, OT supplemented with 10 μM of the broad-spectrum MMP inhibitor GM6001^69^, or OT supplemented with 10 μM of the cysteine protease inhibitor E-64^70^. The selection of inhibitors and dosages was made based on their previous use in cartilage explant^37–39^ and cartilage resorption cultures^36^ as well as their well-established effects on osteoclastic bone resorption^57^.

### Articular cartilage resorption assays

Osteoclast-mediated degradation of bovine articular cartilage was performed as previously described^36^. In brief, bovine articular cartilage was isolated from the femoral condyles of three bovine knees, using a biopsy punch and a scalpel, followed by immersion in liquid nitrogen to render the cartilage metabolically inactive and subsequent fixation of the tissue in 70% ethanol at 4 °C for at least 5 days prior to use. Mature human osteoclasts were generated from CD14^+^ monocytes isolated from the peripheral blood of anonymised blood donors as previously described^71,72^. Osteoclasts were seeded on the cartilage in 96-well culture plates at a density of 1.0 × 10^5^ cells/well and were cultured at 37 °C and 5% CO_2_ in alpha minimum essential medium (αMEM) containing 10% heat-inactivated FBS, 100 units/ml penicillin, 100 µg/ml streptomycin, 388 µg/l thymidine, 25 ng/ml macrophage colony-stimulating factor (M-CSF, R&D Systems, Minneapolis, MN) and 25 ng/ml receptor activator of nuclear factor kappa-B ligand (RANKL, R&D Systems). The medium was supplemented with 5 µM E-64, 10 µM GM6001, 5 µM E-64 and 10 µM GM6001 combined (E-64/GM6001), or a DMSO vehicle (1:2000 in medium), all from Sigma-Aldrich (St. Louis, MO). The selection of inhibitors and dosages was made based on their well-established effects on osteoclastic bone resorption^57^ as well as their previous use in cartilage resorption^36^ and cartilage explant^37–39^ cultures. Cartilage without osteoclasts was cultured as background samples for biomarker measurements. The media were changed by demi-depletion after 24 hours. After an additional 3–4 days the media were collected and stored at −20 °C until analysis. Three independent resorption assays (n = 3–6 per condition) were performed in this study.

### CTX-II

CTX-II released from resorbed cartilage and BEX cultures was measured in the culture supernatants using the Serum Pre-Clinical CartiLaps ELISA (IDS, The Boldons, UK), according to the manufacturer’s instructions. Cartilage resorption supernatants were diluted 1:1–1:8. BEX supernatants were measured for CTX-II at a selection of relevant time points, based on the GPDPLQ_1237_ induction data (days 5, 7, 10, 14, 17, and 21), with samples diluted 1:5–1:500 as needed. Any samples measuring below the detection limit, as specified by the manufacturer, were assigned the detection limit (3.7 pg/ml) as their result. The detection limit was adjusted to 18.5 pg/ml for the BEX samples to compensate for the lowest dilution factor used being 1:5.

### Immunohistochemistry

Knees from young adult (19–21 weeks) female Lewis rats were fixed in 10% formalin for 2 weeks followed by decalcification in 15% EDTA for 7 weeks. The decalcified knees were infiltrated with paraffin using a Tissue-Tek VIP 5 Jr. (Sakura Finetek, Alphen aan den Rijn, The Netherlands), embedded in paraffin, and cut into 5–6 µm thick coronal sections using a HM 360 microtome (Microm International GmbH, Walldorf, Germany). The sections were deparaffinised and subjected to heat-induced epitope retrieval in sodium citrate buffer (10 mM tri-sodium citrate dihydrate, 0.05% Tween 20, pH 6) at 60 °C overnight. The following day the sections were washed in tris-buffered saline (TBS) with 0.1% Tween 20 and 1% Triton X-100 (TBS-TT, used for all wash steps), blocked for endogenous peroxidase activity using 1.2% hydrogen peroxide in 70% ethanol, washed, incubated with blocking solution (0.5% casein sodium salt in TBS-TT) for 20 minutes at room temperature, and incubated with the NB427-5G11-4W3 monoclonal antibody or a negative control mouse antibody (Agilent Technologies, Santa Clara, CA, cat. X0931)—dissolved in blocking solution to 5.1 µg/ml and 15.6 µg/ml respectively—overnight at 4 °C. Then the sections were washed, incubated with an HRP-conjugated secondary antibody (Agilent Technologies, Santa Clara, CA, cat. K4001) for 30 minutes at room temperature, washed, incubated with diaminobenzidine (DAB) chromogen, rinsed in tap water, counterstained with Mayer’s haematoxylin, rinsed and mounted. Digital micrographs of the tibial articular cartilage and growth plate were obtained with an Olympus DP71 digital camera mounted on a BX-60 microscope with 10x and 20x objectives using the Olympus cellSens software (Olympus, Center Valley, PA). The brightness and contrast of each individual image was adjusted for clarity using the Fiji^73^ Brightness/Contrast tool in the automatic mode.

### Statistical analysis

The BEX biomarker data were used to calculate the AUC of each explant. The group AUCs were analysed using the Kruskal-Wallis test with Dunn’s multiple comparisons test against the OT condition. Statistical significance was considered to be P < 0.05. Means ± standard errors of the mean (SEMs) and P-values from the multiple comparisons tests are reported in the results section. Statistical significance is indicated with symbols in the figure where *P < 0.05. Statistical comparisons and plotting of graphs were performed in GraphPad Prism v7.01 for Windows (GraphPad Software, La Jolla, CA). Graphs represent group means and SEMs.

For the statistical analysis of cartilage resorption assays, the background biomarker measurements were subtracted from all other conditions within the same resorption assay, n = 3–6 per condition. The background-subtracted data were normalised to the vehicle condition mean. The mean percentages of each condition from each resorption assay were pooled for analysis (n = 2 for E-64/GM6001 and n = 3 for other conditions, n represents the number of resorption assays the condition was used in), reported as percentages of the vehicle ± SEMs. The data were analysed with a two-tailed one-way analysis of variance (ANOVA), with the assumption that the data were normally distributed, followed by either Tukey (for GPDPLQ_1237_) or Dunnett’s T3 (for CTX-II) post hoc tests depending on whether or not equal variances could be assumed based on Levene’s test. Statistical significance was considered to be P < 0.05. Significance levels from the post hoc tests, relative to the vehicle condition (unless otherwise specified), are reported with P-values in the results section and using symbols in the figure where *P < 0.05 and **P < 0.01. Statistical comparisons were performed using IBM SPSS Statistics v24.0.0.0 64-bit edition for Windows (IBM, Armonk, NY). Graphs were plotted in GraphPad Prism v7.01 for Windows (GraphPad Software, La Jolla, CA) and represent group means and SEMs.

### Ethical approval

Mouse immunisation procedures were approved by the Beijing Administration Office of Laboratory Animal and the animal ethics committee of Nordic Bioscience (Beijing).

The use of peripheral blood from anonymised blood donors, obtained from a blood bank, for osteoclast cultures in this study was covered by the general ethical approval for research use of donor material in accordance with the Transfusion Medicine Standards (TMS) of the Danish Society of Clinical Immunology (DSKI). Informed consent to participate as an anonymised healthy control was obtained from each of the participating donors in writing as part of standard practices at the blood bank at Rigshospitalet, Copenhagen University Hospital, Copenhagen, Denmark. Donors participated in compliance with the Helsinki Declaration.

Human serum, plasma, and urine from OA patients, RA patients, and healthy donors for GPDPLQ_1237_ measurements were commercially sourced.

## Supplementary information

GPDPLQ<sub>1237</sub> Supplementary Info

## Data Availability

The datasets generated during and/or analysed during the current study are available from the corresponding author on reasonable request.
