# Supplementary material for: GPDPLQ1237—A Type II Collagen Neo-Epitope Biomarker of Osteoclast- and Inflammation-Derived Cartilage Degradation in vitro
Source: Sci Rep. 2019 Feb 28;9:3050. doi: 10.1038/s41598-019-39803-0 (PMC6395810; doi:10.1038/s41598-019-39803-0)
Supplement: Supplementary file 1 — GPDPLQ1237 Supplementary Info [file 41598_2019_39803_MOESM1_ESM.pdf]

GPDPLQ$_{1237}$—A Type II Collagen Neo-Epitope Biomarker of Osteoclast- and Inflammation-Derived Cartilage Degradation *in vitro*

Henrik Löfvall[1,2], Anna Katri[1,3], Aneta Dąbrowska[1], Morten A. Karsdal[1], Yunyun Luo[1], Yi He[1], Tina Manon-Jensen[1], Morten H. Dziegiel[4], Anne-Christine Bay-Jensen[1], Christian S. Thudium[1], Kim Henriksen[1]

[1]Nordic Bioscience, Herlev, Denmark.

[2]Division of Molecular Medicine and Gene Therapy, Lund Strategic Center for Stem Cell Biology, Lund, Sweden.

[3]Department of Drug Design and Pharmacology, Copenhagen University, Copenhagen, Denmark.

[4]Department of Clinical Immunology, Rigshospitalet, Copenhagen University Hospital, Copenhagen, Denmark.

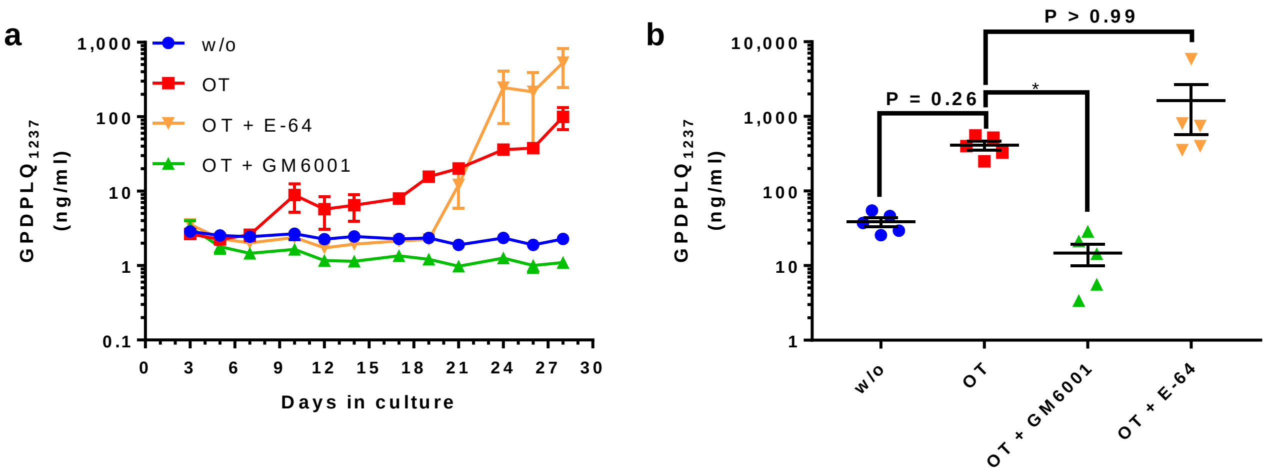

Supplementary Figure S1. GPDPLQ$_{1237}$ release into BEX supernatants upon stimulation and inhibition of MMP activity. The BEX cultures were untreated (w/o), treated with OSM and TNFα (OT) without protease inhibitors, or OT with the protease inhibitors GM6001 (OT + GM6001) or E-64 (OT + E-64). Data from one representative experiment are presented as the mean GPDPLQ$_{1237}$ levels per group at different time points throughout the experiment (a) and as the AUCs of each individual explant (b). Error bars represent the SEMs. Statistical significance levels relative to the OT condition in the AUC data are indicated by *$P < 0.05$.
